# Bedside lung ultrasound versus chest CT in critically ill patients: a cross-sectional diagnostic accuracy study

**DOI:** 10.3389/fmed.2026.1724227

**Published:** 2026-03-31

**Authors:** Ahlem Trifi, Hounaida Galai, Asma Mehdi, Lynda Messaoud, Eya Seghir, Asma Ouhibi, Rochdi Nasri, Sami Abdellatif

**Affiliations:** 1 Medical ICU, Teaching Hospital of la Rabta, Tunis, Tunisia; 2 Faculty of Medicine, Tunis El Manar University, Tunis, Tunisia

**Keywords:** acute respiratory failure, chest computed tomography, critically ill patients, diagnostic accuracy, lung ultrasound

## Abstract

**Study aim:**

Evluate bedside lung ultrasound (LUS) diagnostic accuracy versus chest computed tomography (CT) in adult ICU patients with respiratory distress.

**Design and methods:**

Single-center, cross-sectional study of 200 paired LUS-CT examinations. LUS was performed within 12 h, scanning six zones per hemithorax and classifying findings into A-lines, C1 (pneumonia), C2 (atelectasis), B1, B2 (interstitial patterns), pleural effusion, and emphysema. Diagnostic performance metrics included sensitivity, specificity, predictive values, likelihood ratios, and ROC analysis.

**Key results:**

CT showed consolidations (60.5%), pleural effusions (42.5%), interstitial syndrome (28.5%), ground-glass opacities (26%), and emphysema (23.5%). LUS detected C1 in 66.5% (posterior-inferior predominance), B2 in 40.5%, B1 in 40%, pleural effusions in 38.5%, C2 in 13.5%, and emphysema in 16.5%. Overall concordance with CT was 81%. LUS had very high specificity for pleural effusion (Sp = 96.5%, PLR = 24.5, accuracy = 91.9%) and atelectasis (Sp = 93.7%, NPV = 97.6%, PLR = 12.6, accuracy = 92.3%), very high sensitivity for alveolar-interstitial syndrome (Se = 96.2%, NPV = 98.3%, NLR = 0.05), and good sensitivity for consolidations (Se = 89.3%).

**Conclusion:**

LUS is a reliable bedside tool that can reduce CT use, radiation exposure, and enable dynamic monitoring; structured training is essential.

## Introduction

Acute respiratory failure (ARF) is one of the leading causes of admission to intensive care units (ICUs) (1). Multiple pulmonary conditions may underlie ARF and often coexist in the same patient, including pneumonia, acute pulmonary edema, lesional edema, atelectasis, and pleural effusion (air, fluid, or mixed).

To accurately determine the etiology and assess the degree of lung aeration, chest computed tomography (CT) is considered the gold standard imaging modality. Although CT provides essential diagnostic information in critically ill patients, interpretation can be challenging when pulmonary abnormalities coexist with cardiac overload, intra-alveolar hemorrhage, or other confounding features. Furthermore, the clinical instability of ICU patients often limits CT feasibility in emergency settings, and the use of ionizing radiation remains a significant concern.

Consequently, there is a need for an alternative imaging modality that is simple, non-invasive, non-irradiating, bedside-accessible, and reproducible. In this context, Lung Ultrasound (LUS) has gained increasing attention despite earlier criticism related to the presence of air within alveoli and the interposition of the bony thoracic cage, which were thought to limit its usefulness (2, 3). Over the past decade, the better understanding of lung sonographic semiology and the recognition of meaningful artifacts have led to a growing interest in LUS, particularly in ICU patients (3, 4). The most interesting applications of LUS in ICU patients include: diagnosis of acute respiratory distress syndrome (ARDS) where LUS follows a pattern of aeration to de-aeration and increased lung density. Thus, a number of LUS scoring systems have been proposed to provide a quantitative approach to lung aeration assessment (5, 6). The COVID-19 pandemic further heightened interest in LUS scoring systems as a clinical tool to support direct bedside patient care (7). Also, in ventilation strategies, LUS can identify atelectatic lung guiding recruitment maneuvers and positive end expiratory pressure (PEEP) titration (8). Likewise, to ascertain suitability for extubation, an integrated thoracic ultrasound evaluation encompassing lung, diaphragm, and cardiac sonographic data has been demonstrated to accurately predict post-extubation distress (5). The clinical utility of bedside imaging in ARF has expanded to include severe chest trauma (9, 10).

*Still, the key question remains*: how reliable is lung ultrasound in identifying the spectrum of pulmonary pathologies—such as consolidations, interstitial syndromes, pleural effusions, or pneumothorax—that frequently coexist in ICU patients?

To address this question, we conducted a study aimed at evaluating the diagnostic performance of lung ultrasound in ICU patients presenting with respiratory distress, using chest CT as the reference standard.

## Materials and methods

### Study design, setting, and ethical approval

This was an observational, cross-sectional, single-center study conducted in the medical ICU of La Rabta Hospital, in collaboration with the radiology department, over a 26-month period (November 2022 to December 2024). The study protocol was approved by the local Ethics Committee of La Rabta Hospital (Approval No. 2023-12). All patients or their legal tutors were informed about the study’s purpose and procedures and they provided written informed consent to participate in this study.

### Study population

All adult patients (≥18 years) presenting with respiratory distress—either on admission or during their ICU stay—and requiring a chest CT scan were eligible. Each patient was included only once. Exclusion criteria comprised poor acoustic windows, uncooperative behavior, or a time interval exceeding 12 h between LUS and chest CT.

### Study protocol

A consecutive sampling approach was used. Each eligible patient who underwent a chest CT scan received a lung and cardiac ultrasound examination within 12 h. Ideally, the optimal time interval should be ≤6 h, and obviously, the shorter the interval, the more valid the comparison. Up to a maximum of 12 h is considered acceptable. Beyond 24 h, it is a long interval, meaning that LUS and CT no longer “see” the same lung, and should be avoided as it can generate a major temporal discrepancy bias. The 12-h interval was chosen taking into account the availability of the senior physician experienced in LUS. When the CT scan is performed during the day, the time to perform LUS is optimized to be as short as possible. When it is performed during on-call duty, the interval can be up to 12 h, which is still acceptable for the validity of the results.

Chest CT scans were performed using two types of scanners (General Electric* and Siemens*). The actual resolution and objectively measurable values are very close between these two brands of CT scan. The number of radiologists involved in this study consisted of three radiologists, with professorial and associate professorial status. They were blinded to patient identity and LUS data. However, they were informed by the clinical context and the indication for the chest CT scan. The main CT findings assessed included: ground-glass opacities indicative of alveolar filling, consolidations with or without volume loss (suggesting atelectasis or pneumonia), centrilobular micronodules suggestive of infectious bronchiolitis, pleural effusions (liquid, gaseous, or mixed), and pulmonary embolism. Associated findings such as fibrotic changes, pleural abnormalities, or mediastinal lymphadenopathy were also recorded.

**Figure 1:**
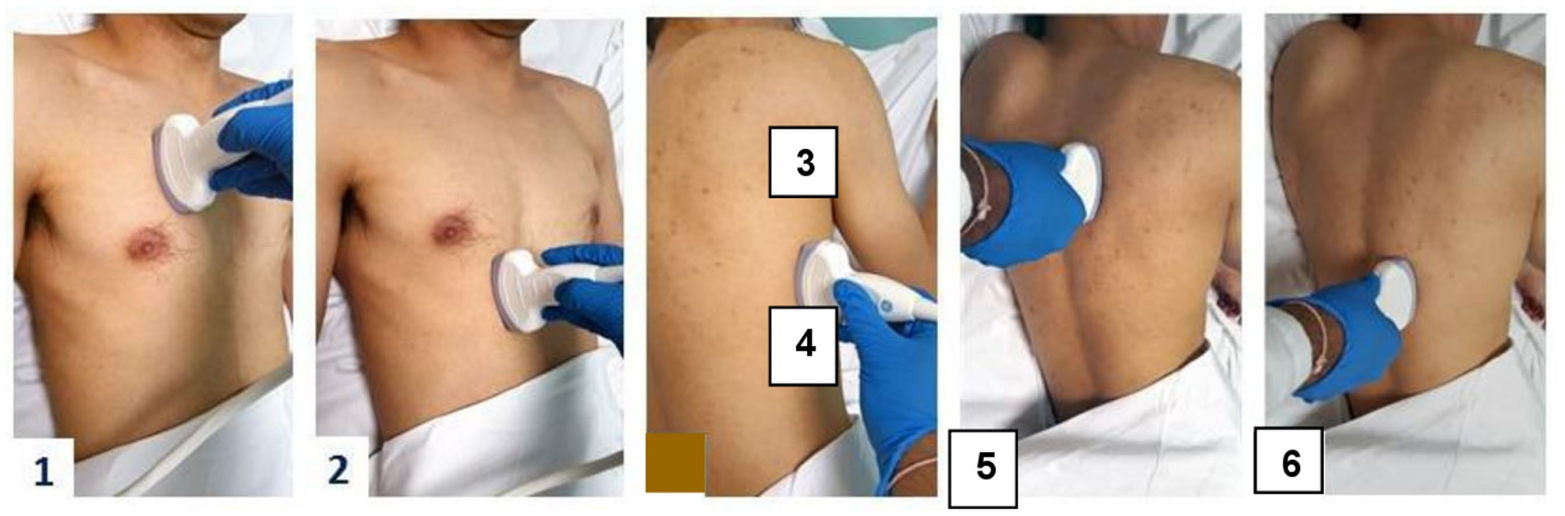
Six-zone/hemithorax approach. For lung ultrasound examination, we chose the approach of six zones per hemithorax. Each hemithorax was divided into three regions: anterior, lateral, and posterior. Each region has an upper and lower quadrant, i.e., six quadrants per hemithorax and twelve quadrants in total.

Lung ultrasound was performed at the bedside with the patient in a semi-recumbent position (30°–45° from supine), using a convex probe with a frequency range of 3–5 MHz to explore deeper lung regions. Examinations were carried out using a MyLab™ SIGMA device (Esaote S.p.A., Genoa, Italy) equipped with a 5 MHz phased-array probe for cardiac evaluation, a 7 MHz linear probe for superficial structures, and a low-frequency convex probe (3–5 MHz) for abdominal and thoracic imaging. Pulsed, continuous, color, and tissue Doppler modes were available. The LUS operator is a professor of intensive care medicine who holds a university diploma in ultrasound in intensive care (specifically thoracic ultrasound, which includes cardiac/lung/ diaphragmatic ultrasound). The certification was awarded by the Faculty of Medicine of Tunis (Tunisia) in 2014 and was obtained after passing theoretical and practical exams, including mini-clinical evaluation exercises, image/video clip reviews by experienced mentors, and a portfolio review. Since receiving this certification, the operator has performed hundreds of chest ultrasound scans. The LUS operator was blinded to both clinical and CT data. A six-zone scanning approach per hemithorax was adopted (Figure 1). This approach was modeled on protocols previously used by validated studies in ICUs (11–13). Sagittal and intercostal orientations were used to optimize image acquisition. Normal lung appearance was characterized by the presence of A-lines in 2D mode and the “seashore sign” in M-mode, indicating normal sliding of the visceral and parietal pleura. B-lines were defined as vertical, hyperechoic artifacts extending from the pleural line, consistent with interstitial syndrome. Based on their number and distribution, two interstitial profiles were identified as B1 profile (sparse B-lines, indicating mild interstitial syndrome), and B2 profile (numerous confluent B-lines, suggestive of alveolar–interstitial involvement). Consolidations appeared as hypoechoic areas with dynamic air bronchograms suggestive of pneumonia (C1 profile), while static bronchograms were more consistent with atelectasis (C2 profile). Ultrasound patterns were interpreted according to the Bedside Lung Ultrasound in Emergency (BLUE) protocol (14).

### Data collection

For each case (defined as a paired CT and ultrasound examination), all imaging findings were recorded. Chest CT served as the reference standard for evaluating the diagnostic performance of lung ultrasound. Demographic and clinical characteristics, indications for CT, ventilatory settings, and hemodynamic parameters were also collected.

### Primary outcome

It was the diagnostic accuracy of lung ultrasound in detecting consolidation, interstitial syndrome, and pleural effusion, compared with chest CT as the gold standard.

### Statistical analysis

Results from CT and ultrasound were analyzed using standard diagnostic accuracy tests, including sensitivity, specificity, positive predictive value (PPV), and negative predictive value (NPV). A “true positive” was defined as a finding identified by lung ultrasound and confirmed on chest CT. Sample size estimation was based on an expected mean prevalence of CT-detected lung disease of 57%, with anticipated sensitivity and specificity values of 92 and 91%, respectively, and a 5% margin of error. The minimum required sample size was calculated at 200 cases.

Diagnostic performance for each type of lung affection was evaluated using receiver operating characteristic (ROC) curves, and the area under the ROC curve (AUC) was calculated. Data were analyzed with SPSS version 20 (SPSS Inc., Chicago, IL, USA). Categorical variables were expressed as percentages and continuous variables as mean ± standard deviation (SD) for normally distributed data or median (interquartile range, IQR 25%–75%) for non-normal distributions. A *p*-value <0.05 was considered statistically significant.

## Results

**Figure 2:**
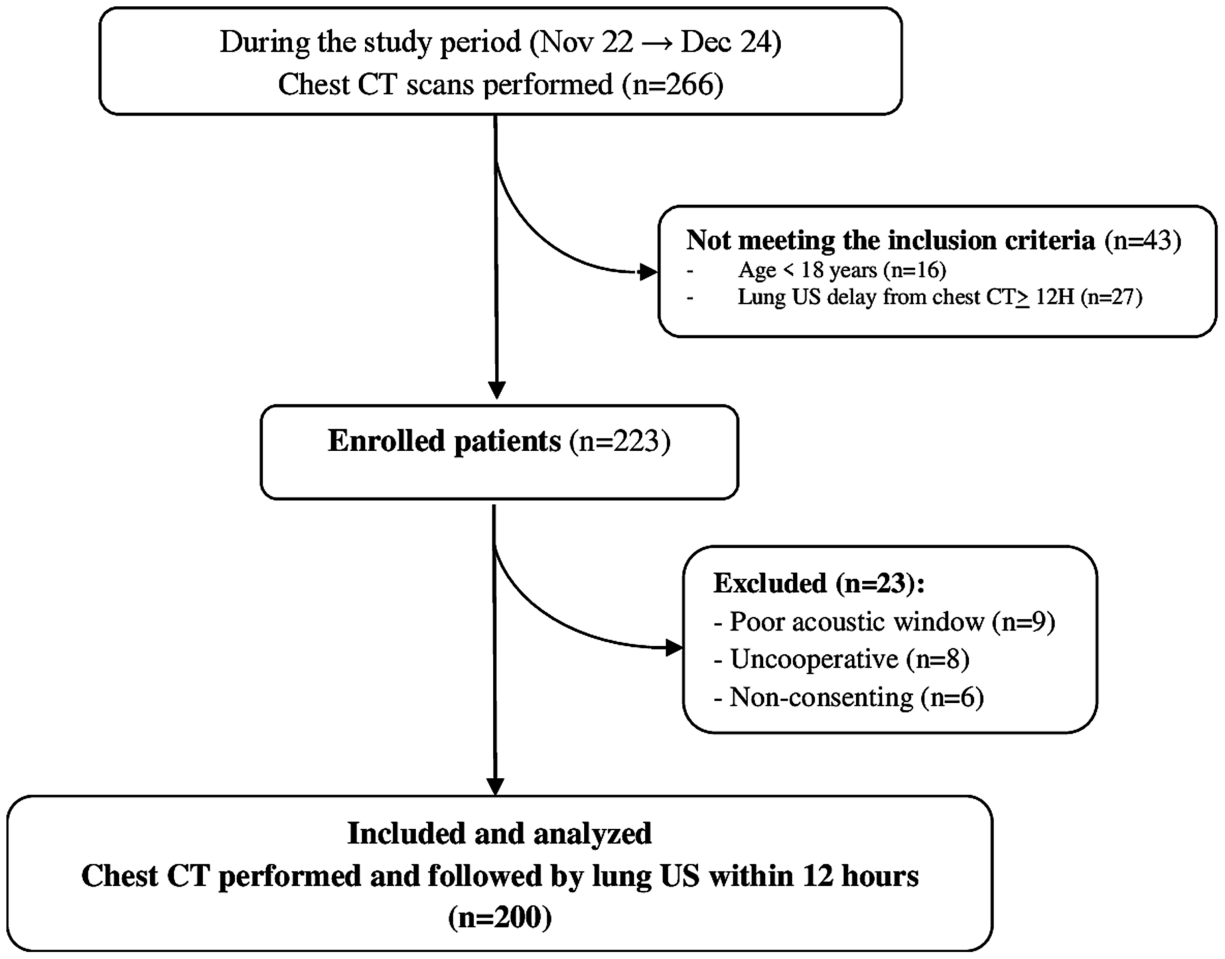
Study flowchart. CT, computed tomography, US, ultrasound.

During the study period, a total of 266 chest CT scans were performed. Among these, 223 patients met the inclusion criteria. After applying the predefined exclusion criteria, 200 cases were retained for analysis (Figure 2). Baseline demographic and clinical characteristics are summarized in Table 1. Chronic respiratory disease was the most frequent medical history (58.5%), including chronic obstructive pulmonary disease (*n* = 48), asthma (*n* = 21), bronchiectasis (*n* = 17), sequelae of previous tuberculosis or SARS-CoV-2 infection (*n* = 11), and diffuse interstitial pneumonia (*n* = 10). Pneumonia investigation was the main indication for chest CT (59.5%). A total of 40.5% of patients were under invasive mechanical ventilation, and transport-related complications occurred in 15 cases.

**Table 1:** Baseline characteristics.

Clinical data	Patients (*n* = 200)
Demographic	
Age, y (med [IQR])	64 [46–73]
Sex-ratio (M/F)	1.7
Origin, *n* (%)	
Emergency department	117 (58.5)
Medical	36 (18)
Surgical	6 (3)
Other	41 (20.5)
Comorbidities, *n* (%)	
Chronic respiratory failure	107 (53.5)
Hypertension	98 (49)
Diabetes	91 (45.5)
Heart disease/arrhythmia	77 (39.5)
Chronic renal failure	37 (18.5)
Dyslipidemia	28 (14)
Reason of admission to ICU, *n* (%)	
Respiratory distress	136 (68%)
Hemodynamic	25 (12.5)
Neurologic	24 (12)
Metabolic	11 (5.5)
Polytrauma	4 (2)
Severity scores, (med [IQR])	
SAPS II	32 [22–44]
APACHE II	13 [10–20]
SOFA	4 [2–6]

Data in regards to chest CT scan practice	Patients (*n* = 200)
Practice time from respiratory signs, d (med [IQR])	1 [1–2]
Indications	
Search for pneumonia	119 (59.5%)
Suspicion of pulmonary embolism	70 (35%)
Suspicion of SARS-CoV2 pneumonia	11 (5.5%)
Conditions of transport, *n* (%)	
Respiratory support	
Nasal goggles	44 (22)
Facial mask	36 (18)
High concentration mask	36 (18)
HNFO	2
NIV	3
IV	81 (40.5)
Vasopressor support	
*N* (%)	16 (8%)
Median posology (mg/H)	1.8 [0.5–3]
Complications of transport *n* (%)	
Increased oxygen requirements	11 (5.5)
Increased vasopressor requirements	3
Accidental fall	1

IQR, interquartile range, ICU, intensive care unit, CT, computed tomography, SAPS, simplified acute physiology score, APACHE, acute physiology and chronic health evaluation, SOFA, sepsis-related organ failure assessment, SARS-CoV, severe acute respiratory syndrome coronavirus, HNFO, high-flow nasal oxygenation, NIVm non-invasive ventilation, IV, invasive ventilation.

The predominant CT abnormalities were pulmonary consolidations (*n* = 121, 60.5%), mainly located in basal regions (87.6%), followed by pleural effusions (*n* = 85, 42.5%) and interstitial syndrome (*n* = 56, 28%). Furthermore, 28 cases (14%) of pulmonary embolism (PE) were identified on the chest CT scan. Their locations and extent and more detailed analysis of CT findings are detailed in Supplementary Table S1. Among these 28 cases of PE, LUS showed a C1 profile (infection-like consolidation) in 11 cases and a C2 profile (atelectasis-like consolidation) in 7 cases.

Lung ultrasound most frequently revealed the C1 profile (pneumonia-like consolidation; *n* = 133, 66.5%), followed by B1 and B2 profiles and pleural effusions, which were bilateral in 71.5% of cases (Table 2). Across the 2,400 explored areas, C1, B1, A, and B2 profiles were identified in comparable proportions, with a predominance of the infectious C1 profile (34.4%), mainly located in posterior-inferior zones (44.6%). The normal A profile was primarily seen in anterior-superior zones (36.5%). The distribution of ultrasound profiles by lung region is shown in Supplementary Table S2.

**Table 2:** Lung US presented by case study.

Lung US profiles	*n* = 200
A profile (normally aerated lung)	No patient had an A profile in all (or most) of the areas explored
B1 profile (minimal/moderate interstitial syndrome)	80 (40%)
B2 profile (alveolar interstitial syndrome)	81 (40.5%)
C1 profile (infection-like consolidation)	133 (66.5%)
C2 profile (atelectasis -like consolidation)	27 (13.5%)
Pleural effusion	77 (38.5%)
Bilateral	50/77 (71.5%)
Unilateral	
Right	18
Left	9
Pneumothorax	2
Right	1
Left	1
Other	
Emphysema	33 (16.5%)
Abscess	5 (2.5%)

US, ultrasound.

Overall, lung ultrasound findings were concordant with chest CT results in 161 cases (81%). The 38 discordant cases are detailed in Supplementary Table S3. The majority of false positives cases were of pneumonia-like consolidation (*n* = 7), which truly corresponded to alveolar hemorrhage on chest CT. False negatives included emphysema, excavation, nodules, and one case of pneumothorax. This discrepancy, while certainly diagnostically important, had no significant clinical impact since the CT scan was the primary basis for clinical decision-making.

By lung condition, the LUS was more specific in identifying pleural effusion (Sp = 96.5%, PPV = 94.8%, NPV = 90.2%, PLR = 24.5, accuracy = 91.9%) and atelectasis (Sp = 93.7%, NPV = 97.6%, PLR = 12.6, accuracy = 92.3%). LUS was more sensitive in diagnosing alveolar-interstitial syndrome (Se = 96.2%, NPV = 98.3%, and NLR = 0.05). Its contribution was modest in diagnosing consolidations, where the best indicator was a sensitivity of 89.3% and NLR = 0.16 (Table 3). All Receiver operating characteristic (ROC) curves for each lung condition, and contingency tables comparing ultrasound and CT findings, are presented in Supplementary material.

**Table 3:** Concordance between lung US and chest CT findings: analysis by lung affection.

US lung affection	AUC/ROC (CI 95% P)	Sensitivity (%) 95% CI	Specificity (%) 95% CI	PPV (%) 95% CI	NPV (%) 95% CI	PLR 95% CI	NLR 95% CI	Accuracy (%) 95% CI
Alveolar-interstitial syndrome	0.875 [0.823–0.926] <10^−3^	96.2 [86.8–99.5]	78.8 [71.3–85]	61.8 [54–68.9]	98.3 [93.6–99.6]	4.53 [3.3–6.22]	0.05 [0.01–0.19]	83.3 [77.4–88.2]
Atelectasis	0.868 [0.763–0.974] <10^−3^	80 [56.4–4.3]	93.7 [88.9–96.8]	59.3 [44–72.8]	97.6 [94.4–99]	12.65 [6.86–23.35]	0.21 [0.09–0.51]	92.3 [87.6–95.6]
Consolidation	0.788 [0.719–0.858] <10^−3^	89.3 [82.3–94.2]	68.4 [56.9–78.4]	81.2 [75.6–85.7]	80.6 [70.8–87.7]	2.82 [2.03–3.92]	0.16 [0.09–0.27]	81 [74.9–86.2]
Emphysema	0.823 [0.738–0.908] <10^−3^	66 [50.7–79.1]	98.7 [95.3–99.8]	94 [79.4–98.4]	90.4 [86.3–93.3]	50.1 [12.5–201.6]	0.34 [0.23–0.51]	90.9 [86–94.5]
Interstitial syndrome	0.743 [0.666–0.821] <10^−3^	75.4 [62.2–85.8]	73.2 [65–80.4]	53.8 [45.9–61.4]	87.9 [81.9–92]	2.81 [2.06–3.85]	0.34 [0.21–0.53]	73.8 [67.1–79.8]
Pleural effusion	0.912 [0.864–0.960] <10^−3^	85.9 [76.6–92.5]	96.5 [91.3–99]	94.8 [87.4–97.9]	90.2 [84.4–93.9]	24.5 [9.3–64.3]	0.15 [0.09–0.25]	91.9 [87.3–95.3]

US, ultrasound, CT, computed tomography, AUC, area under the curve, ROC, receiver operating characteristic curve, CI, confidence interval, PPV, positive predictive value, NPV, negative predictive value, PLR, positive likelihood ratio, NLR, negative likelihood ratio.

## Discussion

In this study, 200 paired chest CT and lung ultrasound (LUS) examinations performed within a 12-h interval were analyzed in 200 ICU patients. We interpret the study performance metrics as follows: high specificity for pleural effusion and atelectasis: (pleural effusion Sp 96.5%, atelectasis Sp = 93.7%) and high positive likelihood ratios indicate that a positive LUS finding for these conditions strongly increases the probability of true disease. This makes LUS particularly useful to rule in effusion or atelectasis at the bedside. High sensitivity for alveolar-interstitial syndrome but very low negative LR that indicate LUS is excellent at ruling out diffuse interstitial involvement when the exam is negative. This supports LUS as a screening tool for interstitial syndromes in the ICU. Good but imperfect performance for consolidations: sensitivity for consolidation (Se = 89.3%) and overall accuracy (~81%) are strong but not perfect. Overall concordance at 81% indicates substantial agreement with CT but leaves room for clinically meaningful discordance in about one in five exams. The pattern of discordance mainly included missed small effusions, central consolidations, or ground glass opacities.

These findings are consistent with prior literature. Indeed, multiple ICU studies and meta analyses have shown LUS outperforms chest radiography and approaches CT for many pathologies, particularly pleural effusion, consolidation abutting the pleura, and interstitial syndrome (15–23). The present results align with that body of evidence and reinforce LUS as a high value bedside modality.

Nevertheless, the accuracy of LUS in identifying consolidations that we found makes a considerable difference if compared to other reported results showing sensitivities >85% and specificities >90% (24–28). In patients with suspected pneumonia or unspecified X ray findings in the emergency department LUS is more sensitive and specific in the diagnosis of pneumonia and is less time-intensive and costly (29).

We may explain these differences by several physical and interpretative limitations. False negatives can occur for deep, centrally located consolidations not abutting the pleura or when acoustic windows are limited. Clinicians should therefore interpret a negative LUS cautiously when clinical suspicion for focal consolidation remains high. Posterior-inferior predominance of C1 findings aligns with expected gravity dependent consolidation patterns in ICU patients. LUS is a reliable tool for detecting postoperative or anesthesia-related atelectasis (30–32). It is important to note that diagnostic categories (C1 vs. C2) rely on pattern recognition that is operator-dependent. In this work, we did not report on inter-observer agreement because the acquisition and interpretation of the images were performed by the same clinician (LUS operator) who was blinded for clinical and CT scan data as he was off of clinical activity towards the patients included in this study.

In our series, fluid pleural effusion was diagnosed by LUS in 38.5% of cases with a high diagnostic performance (AUC/ROC = 0.912 [0.864–0.960], and sensitivity = 85.9%). This finding aligns with that of Safai et al. (33), and to those of a mixed medical-surgical ICU study (sensitivity = 89.7% and specificity = 94.1%) (34). Similarly, Ali et al. reported an excellent diagnostic accuracy for LUS in pleural effusion detection (96%) (35). LUS has also been shown to distinguish different types of effusions based on echogenicity, internal structure, and thickness, helping identify complicated effusions with septations or fibrin strands and estimating fluid volume for drainage (36–38). LUS performs better than chest X in demonstrating pleural effusions (sensitivity = 93% vs. 43%) and identified significantly smaller volumes of effusion (39). Regarding pneumothorax, it was rarely identifiable in our study (2 cases by LUS and CT identified 3 pneumothoraces, resulting in one false negative case). Although the small number of events, our results are consistent with those of Sosa et al. (34), who reported high specificity (98.1%) and sensitivity (100%) for LUS in pneumothorax detection. The “absence of lung sliding” sign showed a sensitivity of 87.2% and specificity of 99.4%, while the “lung point” sign demonstrated 82.1% sensitivity and 100% specificity (40). The difficulty in diagnosing pleural air effusion by LUS is reasonable in critical patients because of the hyperinflated lungs in several ICU situations (emphysematous patients, bronchospasm with gas trapping, endobronchial intubation) (3). Data on LUS performance for emphysema remain limited, likely due to poor US wave transmission through hyperaerated parenchyma.

To summarize, LUS is a practical tool to reduce CT dependence and enable dynamic bedside assessment. It can be used for rapid triage, to guide immediate interventions (e.g., drainage of effusion), and for serial monitoring of disease progression or response to therapy. It may reduce CT use lowers radiation exposure and avoids risky intrahospital transport of unstable patients. Nonetheless, implementing LUS requires protocols for when CT remains necessary (e.g., suspected central embolic disease, complex pleuropulmonary anatomy, or when LUS windows are inadequate). Moreover, diagnostic accuracy of LUS is strongly linked to operator skill in both acquisition and interpretation. Programs should include hands on supervised scanning, competency assessment, and periodic quality audits. Simulation and image review rounds can improve consistency.

Otherwise, it is important to emphasize that pre-existing lung disease such as COPD, ILD, or TB sequelae may reduce the specificity of LUS. Although in our series there were 69 patients having these chronic structural changes [COPD (*n* = 48), tuberculosis or SARS-CoV-2 sequelae (*n* = 11), and ILD (*n* = 10)], it is possible that they were wrongly diagnosed as acute pathological findings leading sometimes to discordant cases. Since the presence of the underlying pulmonary condition was known beforehand, it would have been more reasonable to consider these findings as non-specific. While LUS remains sensitive for detecting abnormalities; its diagnostic accuracy for defining the underlying etiology is lower in these patients compared with CT.

Several limitations and unanswered questions should be acknowledged. First, Single center sampling may limit generalizability across ICUs with different case mixes, equipment, or operator experience. Second, the use of two types of chest CT scans could potentially introduce bias in the interpretation of the results, since the CT scan is the reference standard. This bias could be mitigated by the fact that images texture, sound, contrast, and artifact management are adjusted by reconstruction algorithms to guarantee stable and similar image quality. Also, the protocol settings, patient optimization, and post-processing that play a more significant role than the brand alone are standardized in our radiology department. Hence, if there are differences they are often subtle and not remarkable discrepancies in diagnosis. Third, there is a possibility of temporal discrepancy bias and therefore a lung status change due to the chosen 12-h LUS–CT interval. This interval should preferably be ≤6 h, but 12 h is also acceptable. This factor could lead to a false decrease in LUS sensitivity or specificity not because of poor LUS performance, but because the lesion has changed. Finally, the operator dependence and the luck of interobserver agreement review must be recognized. In fact, all LUS examinations were performed by the same trained operator with significant experience in critical care ultrasound, using a predefined and standardized protocol. While this design improves internal consistency by reducing heterogeneity, we recognize that it limits the evaluation of reproducibility and therefore represents a limitation of the study.

We conclude that LUS represents a powerful monitoring and diagnostic tool for critically ill patients. But safe implementation requires structured training, awareness of limitations (central lesions, operator dependence), and clear protocols for when CT remains indicated.

In light of our study and the literature review, we recommend for clinicians to integrate LUS into ICU diagnostic pathways for initial assessment and monitoring of pleural effusion, interstitial syndrome, and peripheral consolidation, while reserving CT for cases with high clinical suspicion despite negative LUS, complex anatomy, or when additional thoracic detail is required. Ensure structured training and ongoing competency assessment.

Future studies should report blinding procedures, interobserver agreement, and a detailed analysis of discordant cases. Multicenter trials would improve generalizability and help define standardized scanning protocols and thresholds for when CT remains necessary. Comparative cost effectiveness analyses and impact on patient centered outcomes (length of stay, complications from transport, radiation exposure) would strengthen the case for broader LUS adoption.

## Data Availability

The original contributions presented in the study are included in the article/Supplementary material, further inquiries can be directed to the corresponding author.
