# Supplementary material for: Bedside lung ultrasound versus chest CT in critically ill patients: a cross-sectional diagnostic accuracy study
Source: Front Med (Lausanne). 2026 Mar 31;13:1724227. doi: 10.3389/fmed.2026.1724227 (PMC13076476; doi:10.3389/fmed.2026.1724227)
Supplement: Supplementary file 1 [file Table_1.DOCX]

**Table 1. Detailed analysis of CT findings**

| Chest CT findings | Studied cases (n=200) |
| --- | --- |
| Consolidation, n (%)  • Region  o Apical  o Basal  o Apico-basal   - Bilateral | **121 (60,5%)**  11  106/121 (87,6)  7  68 |
| Ground glass, n (%)  • Region  o Diffuse throughout the lung  • Bilateral location | **52 (26%)**  25  42 |
| Interstitial syndrome, n (%)   - Bilateral | **57 (28,5%)**  44 |
| Fluid Pleural Effusion, n (%)  • Bilateral  • Unilateral  o Right  o Left  • Abundance   - - Low   - Medium   - High | **85 (42,5%)**  57  28  19  9  35  41  7 |
| Pneumothorax   - Location   o Right  o Left | **3**  2  1 |
| Pulmonary Embolism  • Location  o Right  o Left  o Bilateral   - Arterial topography   - Proximal   - Segmental   - Subsegmental | **28 (14%)**  9  8  11  13  17  14 |
| Atelectasis  • Basal Region   - Bilateral Location | **20 (10%)**  20  12 |
| Emphysema | **47 (23,5%)** |
| Excavation | **2** |

**Table 2. Distribution of A, B, C profiles in the different explored areas by lung US**

|  | Results by area explored  (n=2400) |
| --- | --- |
| A Profile (normally ventilated lung)   - Most affected areas | **754 (31,4%)**  Antero-sup : 275/754 (36,5%)  Antero-inf : 216 (28,7%)  Latéro-sup : 123 (16,3%) |
| B1 Profile (Minimal/Moderate Interstitial Sd)   - Most Affected Areas | **758 (31,6%)**  Latero-sup : 180 (23,8%)  Postero-sup : 150 (20%)  Latero-inf : 131 (17,3) |
| B2 Profile (alvéolar-interstitiel Syndrome)   - Most Affected Areas | **763 (31%)**  Postero-inf: 200 (27%)  Latero-inf: 176 (23,7%)  Postero-sup: 160 (21,5%) |
| C1 Profile (infection-like consolidation)   - Most Affected Areas | **826 (34,4%)**  Postero-inf: 369 (44,6%)  Latero-inf: 215 (26%)  Postero-sup: 165 (20%) |
| C2 Profile (atelectasis -like consolidation)   - - Most Affected Areas | **77 (3,2%)**  Postero-inf : 33 (42,8%)  Latero-inf : 21 (27,3%)  Postero-sup : 13 (17%) |
| Pleural effusion   - Most Affected Areas | **308 (13%)**  Postero-inf: 120 (40%)  Latero-inf: 88 (28,5%)  Postero-sup: 67 (21,7%) |
| Pneumothorax   - Most Affected Areas | **4**  Antero-inf droit (1)  Postero-sup dt (1)  Antero-sup gche (1)  Latero-sup gche (1) |
| Others :   - Emphysema   - Most Affected Areas - Abscess   - Most Affected Areas | **176 (7,4%)**  Antero-inf : 41 (23,3%)  Antero-sup : 37 (21%)  Latero-sup : 34 (19,4%)  **4**  Latero-inf (2)  Postero-inf (2)  Postero-sup (1) |

**Table 3. Cases of discordance between lung US and chest CT data (n=38)**

| Lung Ultrasound | Chest CT scan | N cases |
| --- | --- | --- |
| B2 Profile | Bronchial dilatation | 6 |
| C1 Profile (infectious consolidation) | Ground glass/alveolar hemorrhage | 7 |
| C2 Profile (atelectasis) | Localized pneumonia  Pulmonary infarction | 3  2 |
| A or B1 Profile | Pulmonary nodules  Emphysema  Pneumothorax | 4  8  1 |
| Abscess  Overload lung | Excavation / Nodule/ pneumonia  Alveolar hemorrhage | 5  2 |

**Figure 1. Performance of Lung US in the diagnosis of condensation (pneumonia type)**

|  | | Consolidation CT | |  |
| --- | --- | --- | --- | --- |
|  |  | Yes | No |  |
| Consolidation US | Yes | 108 | 25 | 133 |
|  | No | 13 | 54 | 67 |
|  |  | 121 | 79 | 200 |

1-Specificity

**Sensitivity**

**Figure 2. Performance of lung US in the diagnosis of interstitial syndrome**

**Sensitivity**

|  | | Interst Sd CT | |  |
| --- | --- | --- | --- | --- |
|  |  | Yes | No |  |
| Interst US | Yes | 43 | 37 | 80 |
|  | No | 14 | 101 | 115 |
|  |  | 57 | 138 | 195 |

1-Specificity

**Figure 3. Performance of lung US in the diagnosis of alveolar-interstitial syndrome**

|  | | Alv-Interst Sd CT | |  |
| --- | --- | --- | --- | --- |
|  |  | Yes | No |  |
| Alv-Interst Sd US | Yes | 50 | 31 | 81 |
|  | No | 2 | 115 | 117 |
|  |  | 52 | 146 | 198 |

1-Specificity

**Sensitivity**

**Figure 4. Performance of lung US in the diagnosis of pleural effusion**

|  | | Pl effusion CT | |  |
| --- | --- | --- | --- | --- |
|  |  | Yes | No |  |
| Pl effusion US | Yes | 73 | 4 | 77 |
|  | No | 12 | 110 | 122 |
|  |  | 85 | 114 | 199 |

1-Specificity

**Sensitivity**

**Figure 5. Performance of lung US in the diagnosis of atelectasis**

1-Specificity

|  | | Atelectasis CT | |  |
| --- | --- | --- | --- | --- |
|  |  | Yes | No |  |
| Atelectasis US | Yes | 16 | 11 | 27 |
|  | No | 4 | 163 | 167 |
|  |  | 20 | 174 | 194 |

**Sensitivity**

**Figure 6. Performance of lung US in the diagnosis of emphysema**

|  | | Emphysema CT | |  |
| --- | --- | --- | --- | --- |
|  |  | Yes | No |  |
| Emphysema US | Yes | 31 | 2 | 33 |
|  | No | 16 | 150 | 166 |
|  |  | 47 | 152 | 199 |

**Sensitivity**

1-Specificity
